# Laparoscopic transperitoneal and retroperitoneal adrenalectomy: a 20-year, single-institution experience with an analysis of the learning curve and tumor size [lap transper and retroper adrenalectomy]

**DOI:** 10.1007/s00464-019-07337-1

**Published:** 2020-01-17

**Authors:** Aurel Ottlakan, Attila Paszt, Zsolt Simonka, Szabolcs Abraham, Bernadett Borda, Marton Vas, Bela Teleky, Adam Balogh, Gyorgy Lazar

**Affiliations:** 1 Department of Surgery, Albert Szent-Györgyi Health Center, University of Szeged, Semmelweis u. 8, Szeged, 6720, 6725 Hungary; 2 Department of Surgery, University of Vienna, Währinger Gürtel 18-20, 1090 Vienna, Austria

**Keywords:** Minimally invasive, Adrenalectomy, Retroperitoneal, Transperitoneal, Learning curve, Extra-large tumor

## Abstract

**Background:**

The superiority of laparoscopic transperitoneal (TP) versus retroperitoneal (RP) adrenalectomy is an ongoing debate.

**Methods:**

Data from 163 patients (TP: *n* = 135; RP: *n* = 28) undergoing minimally invasive adrenalectomy were analyzed. Both operative [intraoperative blood loss, previous abdominal surgery, conversion rate, operative time and tumor size] and perioperative [BMI (body mass index), ASA (American Society of Anesthesiologists) score, time of hospitalization, time of oral intake, histology and postoperative complications] parameters were compared. Both the learning curve (LC) and tumor size were analyzed.

**Results:**

We found significant differences in the mean operative time (*p* = 0.019) and rate of previous abdominal surgery (*p* = 0.038) in favor of TP. Significantly larger tumors were removed with TP (*p* = 0.018). Conversion rates showed no significant difference (*p* = 0.257). Also, no significant differences were noted for time of hospitalization, intraoperative blood loss and postoperative complications. In terms of the LC, we saw significant differences in previous abdominal surgery (*p* = 0.015), conversion rate (*p* = 0.011) and operative time (*p* = 0.023) in favor of TP. Large (LT) and extra-large tumors (ELT) were involved in 47 lesions (LT: 40 vs. ELT: 7), with a mean tumor size of 71.85 and 141.57 mm, respectively. Mean intraoperative blood loss was 64.47 ml vs. 71.85 ml, time of hospitalization was 5.10 vs. 4.57 days and mean operative time was 76.52 vs. 79.28 min for LT and ELT, respectively.

**Conclusion:**

A shorter operative time and lower conversion rate in favor of TP were noted during the learning curve. TP proved to be more effective in the removal of large-, extra-large and malignant lesions. The RP approach was feasible for smaller, benign lesions, with a more prolonged learning curve.

Since the introduction of laparoscopic transperitoneal adrenalectomy by Gagner et al. in 1992, several studies have confirmed the advantages of the minimally invasive approach over open adrenalectomy [1]. Minimally invasive adrenalectomy results in a shorter hospital stay and reduced operative time with a decreased rate of perioperative morbidity and mortality. Shortly after the first laparoscopic transperitoneal adrenalectomy, Mercan et al. performed the first endoscopic retroperitoneal adrenalectomy [2] using the posterior approach. The posterior approach has been widely used by Walz et al. and the approach was described as having the main advantage of a direct access to the adrenal glands without interfering with the intraperitoneal organs [3, 4]. In a joint publication by Walz and a South Korean working group, they concluded that the two methods show similarities in some parameters [intraoperative blood loss, tumor size and body mass index (BMI)], although in terms of time of oral intake and operative time, they prefer the posterior retroperitoneal (RP) approach [5, 6]. The greatest among many advantages of the TP method are the safe and simple manipulation during intraoperative laparoscopy and the rapid ligation of the adrenal veins to block catecholamine release. According to some authors, the disadvantages of TP include prolonged operative times even after the learning curve (LC) [7]. When it was first used, the minimally invasive method was reserved for benign, small or moderate-sized adrenal lesions; however, with improved laparoscopic techniques and instrumentation, large (6–10 cm), giant (> 10 cm) and even malignant lesions have been removed with accurate oncological radicality [8, 9]. There is an ongoing debate on the superiority of either TP or RP, which is considerably influenced by the surgeon’s preference. The TP approach may be more feasible for laparoscopic surgeons with its familiar anatomy and wider surgical space [10–13], even though possible adhesions after multiple abdominal procedures may lead to difficulties during laparoscopic manipulation, possibly resulting in an increased rate of conversions. According to a recent work by Chen et al., in terms of perioperative factors, ASA (American Society of Anesthesiologists) scores of 3 or 4 and diabetes were independently associated with perioperative complications, while the presence of pheochromocytomas and tumors over 6 cm in size resulted in the highest rate of complications [14]. In the RP approach, the adrenals are directly accessible, without the incursion of intraperitoneal organs, possibly leading to a lower occurrence of postoperative ileus [5]. In recent years, several meta-analyses have compared the two approaches [10, 11] with mixed results. Our aim was to compare the two methods performed during a 20-year period at the same institution, taking operative and perioperative factors into account, with special consideration for learning curves and tumor size.

## Materials and methods

Perioperative and mid-term results of minimally invasive adrenalectomy were analyzed between January 1998 and April 2018 at the University of Szeged, Department of Surgery. The retrospective investigation, collection of patient data and ethical approval were granted by the Human Investigation Review Board of the University of Szeged (No. 4485). Minimally invasive procedures were carried out in 163 patients (135 TP and 28 RP procedures). Baseline characteristics (male–female ratio, mean age and BMI) showed no significant difference; thus, the two patient groups were considered homogenous with regard to these characteristics (Table 1). The two approaches were compared in terms of previous abdominal surgery, conversion rate, operative time, intraoperative blood loss, tumor size, histology, time of hospitalization, and early and late postoperative complications. The early postoperative period was defined as the in-patient stay, while the late postoperative period lasted from Month 12 to Month 24 (12 + 9 months). Resected lesions were grouped by size as large tumors (LT: 6–10 cm) or extra-large tumors (ELT: > 10 cm). The two groups were analyzed according to tumor size, histology, complete (R0) resection rate in malignant cases, previous abdominal surgery, conversion rate, intraoperative blood loss, time of hospitalization, operative time, BMI and ASA.

**Table 1:** Baseline characteristics for TP and RP

	TP (*n* = 135) mean ± SD	RP (*n* = 28) mean ± SD
Males/females	40/95	9/19
Age (years)	57.15 ± 16.7	47.05 ± 27.8
ASA		
1	3 (2.22%)	0 (0%)
2	51 (37.77%)	6 (23%)
3	57 (42.22%)	17 (58%)
4	24 (17.77%)	5 (19%)
BMI (kg/m^2^)		
Mean	26.09 ± 2.57	25.19 ± 1.3
≤ 25	35 (25.92%)	5 (17.85%)
25–30	62 (45.92%)	13 (46.42%)
30 ≤	38 (28.14%)	10 (35.71%)

*TP* transperitoneal, *RP* retroperitoneal, *ASA* American Society of Anesthesiologists, *BMI* body mass index

Taking into consideration that significantly fewer procedures were performed using the RP approach, the perioperative parameters of a learning curve (28 procedures for each method) were also compared.

Both TP and RP procedures were carried out by two senior endocrine surgeons with several years of experience in both open and minimally invasive adrenalectomies; thus, operative- and perioperative results were reasonably comparable.

### Operative techniques

#### Laparoscopic transperitoneal adrenalectomy

Surgeries were carried out under general anesthesia. A semilateral positioning (on the left or right side) of the patient was used. By angulating the operating table, the highest level of distance was maintained between the iliac crest and the lower ribs. Three or four utility ports were used during the procedure. After dissection of the adrenal gland using either LigaSure or a Harmonic Scalpel and clipping of the vasculature, the specimen was retrieved in an Endobag. Three extra-large lesions (> 10 cm) were retrieved en bloc through an additional Pfannenstiel incision.

#### Laparoscopic retroperitoneal adrenalectomy

Surgeries were carried out under general anesthesia. Patients were placed in prone position. 3 ports were inserted during the procedure. After blunt dissection of the lumbar musculature and Gerota’s fascia, the retroperitoneum was insufflated. Additional trocars were placed under finger supervision. Tissue dissection and handling of vessels were carried out by Ultracision and clipping. The fully dissected adrenal was then separated from the upper pole of the kidney. The specimen was retrieved in an Endobag [15].

### Statistical analysis

Taking the normal distribution of the population into consideration, the *t* test and ANOVA were used. The χ^2^ test and Fischer’s test were employed for categorical variables. A value of *p* < 0.05 was considered significant. Statistical analysis was carried out using SPSS 25.

## Results

During the study period, 135 TP and 28 RP adrenalectomies were performed. Baseline characteristics are shown in Table 1. Even though the rate of previous abdominal surgery was significantly higher for TP [TP: 61 (45.18%) vs. RP: 4 (14%); *p* = 0.038], the conversion rate was considerably (however, not significantly) higher for RP [TP: 6 (4.44%) vs. RP: 5 (18%); *p* = 0.257] (Table 2). In terms of operative time, the TP approach proved to be significantly shorter [TP: 78.51 ± 12.38 min vs. RP: 134.5 ± 12.4 min; *p* = 0.019] with the removal of significantly larger tumors [TP: 56.29 ± 9.02 mm vs. RP: 34.8 ± 11.2 mm; *p* = 0.018]. 40 large and 7 extra-large tumors were resected during the study. Mean tumor size was 71.85 mm and 141.57 mm for large and extra-large tumors, respectively. There were 25 adenomas, 4 metastases, 4 myelolipomas, 2 hyperplasias, 2 pheochromocytomas, 1 vascular malformation, 1 pseudocyst and 1 adrenocortical carcinoma in the large tumor group. Complete (R0) resections were successfully carried out in all malignant lesion cases (4 metastases and 1 adrenocortical carcinoma). Histology in cases involving extra-large tumors confirmed 3 adrenocortical carcinomas, 1 pheochromocytoma, 1 myelolipoma, 1 neurofibroma and 1 cyst. All three adrenocortical carcinomas proved to be R0 resections. Among the 47 patients, previous abdominal surgery occurred in 12 (30.0%) and 5 (71.42%) cases, laparoscopy was performed in 36 (90.0%) and 7 (100%) patients, mean intraoperative blood loss was 64.47 and 71.85 ml, mean time of hospitalization was 5.10 and 4.57 days, operative time was 76.52 and 79.28 min, mean BMI was 23.45 and 27.87, and mean ASA was 2.62 and 2.42 for large- and extra-large tumors, respectively. There was no significant correlation between removed large tumors/extra-large tumors and duration of surgery.

**Table 2:** Operative factors for TP and RP

	TP (*n* = 135)	RP (*n* = 28)	*p* value
Previous abdominal surgery	61 (45.18%)	4 (14%)	0.038
Conversion rate	6 (4.44%)	5 (18%)	0.257
Intraoperative blood loss (ml)	65.7 ± 8.45	50.2 ± 10.78	0.147
Tumor size (mean) (mm)	56.29 ± 9.02	34.8 ± 11.2	0.018
Time of hospitalization (days)	4.25 ± 1.58	4.61 ± 2.24	0.237
Operative time (minutes)	78.51 ± 12.38	134.5 ± 12.4	0.019

*TP* transperitoneal, *RP* retroperitoneal

Considering all cases (*n* = 163), time of hospitalization [TP: 4.25 ± 1.58 vs. RP: 4.61 ± 2.24; *p* = 0.237] and intraoperative blood loss [65.7 ± 8.45 vs. 50.2 ± 10.78; *p* = 0.147] showed no significant difference between the two methods (Table 2).

In terms of perioperative analgesia, the administration of NSAIDs (Nonsteroidal Anti-inflammatory Drugs), such as paracetamol, diclofenac and metamizole sodium, proved to be sufficient. Oral intake was started on Postoperative Day 1 in both groups.

Adenoma was found in 65.92% (*n* = 89) and 64.3% (*n* = 18) of the TP and RP cases, respectively. Other lesions showed the following pattern in TP cases: metastasis: 8.14% (*n* = 11); cyst: 5.18% (*n* = 7); adrenocortical carcinoma: 4.44% (*n* = 6); myelolipoma: 3.7% (*n* = 5); hyperplasia: 3.7% (*n* = 5); vascular malformation: 0.74% (*n* = 1); neurofibroma: 0.74% (*n* = 1) and leiomyosarcoma: 0.74% (*n* = 1). Similar findings were not noted for RP (Table 3). Pheochromocytoma occurred in 6.66% (*n* = 9) and 17.8% (*n* = 5) of TP and RP cases, respectively. Altogether, 18 malignant lesions were confirmed in TP cases (11 metastases, 6 adrenocortical carcinomas and 1 leiomyosarcoma). Complete (R0) resection was confirmed in all cases. Early complications occurred in 5 TP cases (splenic injury, fever, postoperative bleeding, severe hypokalemia and ventricular fibrillation) and 4 RP cases (2 cases of postoperative bleeding and 2 cases of severe wound infection) (Table 4). In terms of late onset complications, only one case (1/135; 0.74%) of postoperative abdominal hernia occurred following TP (Table 4).

**Table 3:** Histology patterns for TP and RP

	TP (*n* = 135)	RP (*n* = 28)
Adenomas	89 (65.92%)	18 (64.3%)
Adrenocortical cc	6 (4.44%)	–
Leiomyosarcoma	1 (0.74%)	–
Metastasis	11 (8.14%)	–
Pheochromocytomas	9 (6.66%)	5 (17.8%)
Cysts	7 (5.18%)	3 (10.7%)
Hyperplasia	5 (3.7%)	2 (7.1%)
Vascular malformation	1 (0.74%)	–
Neurofibromas	1 (0.74%)	–
Myelolipomas	5 (3.7%)	–

*TP* transperitoneal, *RP* retroperitoneal

**Table 4:** Rate of early and late onset complications

	TP *n* = 135	RP *n* = 28
Early complications 1–2 days		
Splenic injury	1	0
Fever	1	0
Intraoperative bleeding	1	2
Severe hyperkalemia	1	0
Ventricular fibrillation (death)	1	0
Severe wound infection	0	2
Late complications 12–21 months		
Postoperative abdominal hernia	1	0

*TP* transperitoneal, *RP* retroperitoneal

**Fig. 1:**
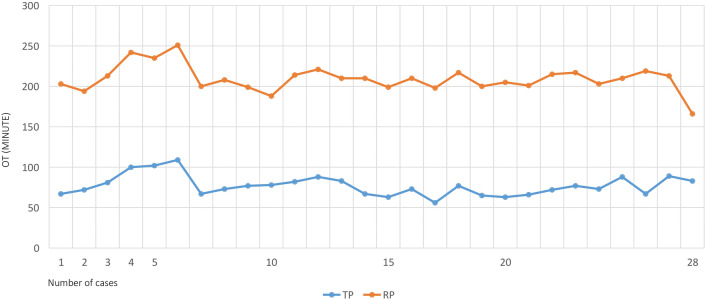
Learning curves (LC) for transperitoneal (TP) and retroperitoneal (RP) procedures for the first 28 cases. *OT* operative time

Considering the notable difference between the number of patients in both groups, outcomes for the first 28 procedures in each group were also analyzed. During the LC, a significant difference was noted in terms of previous abdominal surgery [TP: 10 (35.7%) vs. RP: 4 (14%); *p* = 0.015], conversion rate [TP: 0 vs. RP: 5 (18%); *p* = 0.011] and operative time [TP: 110 ± 8.1 min vs. RP: 134.5 ± 12.4 min; *p* = 0.023], both in favor of the TP approach. In terms of operative time, a similar balance was observed during both LCs (Fig. 1), with significantly shorter operative time and significantly larger tumors removed in the TP group (TP: 52.2 ± 4.8 mm vs. RP: 34.8 ± 11.2 mm; *p* = 0.068). There was no significant difference in terms of intraoperative blood loss or time of hospitalization during the LC (Table 5).

**Table 5:** Comparison of the learning curve for TP and RP

	TP (*n* = 28)	RP (*n* = 28)	*p* value
Previous abdominal surgery	10 (35.7%)	4 (14%)	0.015
Conversion rate	0 (0%)	5 (18%)	0.011
Intraoperative blood loss (ml)	58.2 ± 7.3	50.2 ± 10.78	0.235
Tumor size (mean) (mm)	52.2 ± 4.8	34.8 ± 11.2	0.068
Time of hospitalization	4.12	4.61	0.215
Operative time (mean) (minutes)	110 ± 8.1	134.5 ± 12.4	0.023

*TP* transperitoneal, *RP* retroperitoneal

The learning curve was analyzed during the first 28 cases in both groups, during which a decreasing trend was confirmed with each method (Fig. 1). In the first 10 procedures, both methods showed a considerable decrease and similar patterns of operative time, with notably longer operative times in RP cases (Fig. 1).

## Discussion

In recent years, surgery for adrenal lesions has gradually evolved from the open technique toward the minimally invasive method [16–18]. Laparoscopy carries many advantages compared to the traditional approach, such as shorter operative times, reduced postoperative pain, decreased rate of perioperative and long-term complications, and a reduced hospital stay [1, 19]. After it was introduced by Gagner et al., laparoscopic adrenalectomy became the gold standard for the surgical management of adrenal lesions [1]. Laparoscopic transperitoneal and retroperitoneal approaches are undoubtedly the two most popular methods. In the past decade, both have been in the focus of numerous retrospective studies and meta-analyses, which reported no significant difference in terms of operative outcomes (intraoperative blood loss and operative time) [7, 19, 20].

The results of our retrospective study indicate a shorter operative time and removal of larger tumors for TP. In terms of other parameters, such as time of hospitalization, intraoperative blood loss and perioperative complications, no significant difference occurred between the two approaches.

According to relevant literature data, the three key factors impacting operative time and the rate of conversions include obesity (BMI: > 24 kg/m^2^), tumors larger than 5 cm and the presence of pheochromocytomas [5, 21]. The retroperitoneal approach seems to be less effective in patients with increased BMI due to the relatively thick lumbar subcutaneous layer, which leads to less effective port placement and, thus, hampered and unsafe retroperitoneal manipulation. Although Zonca et al. claimed that RP is safe and effective even for patients with BMI values over 40 [22], most publications recommend TP for obese (BMI: > 30) patients [5].

Obesity may increase operative times as an independent predictive factor; however, in terms of BMI, similar results were obtained between the two groups. Among the 163 patients, 38 (28.14%) and 10 (35.71%) had a BMI ≥ 30 in cases of TP and RP, respectively (BMI: ≤ 25—TP vs. RP: *n* = 35 vs. *n* = 5; BMI: 25–30—TP vs. RP: *n* = 62 vs. *n* = 13; BMI: ≥ 30—TP vs. RP: *n* = 38 vs. *n* = 10) (Table 1.). Thus, no significant difference was noted, although high BMI values may have contributed to the prolonged LC in RP.

Malignant, hormonally active and large (6–10 cm) tumors were previously reserved for open surgery [7]. Conversely, our results confirmed that TP was indeed successful for large lesions (mean tumor size: 56.29 ± 9.02 mm), probably due to the wider operative working field and improved visualization.

The incidence of large, 6–10 cm adrenal tumors is 8.6–38.6%, showing malignancy in a quarter of the cases [23]. Tumor size shows direct correlation with chances of malignancy, although size is not always a reliable predictive factor. Even the most frequently occurring adenomas are often larger than 6 cm; hence, preoperative diagnostics [contrast-enhanced computed tomography (CT) and magnetic resonance imaging (MRI)] have a major impact on minimally invasive adrenalectomy and may suggest local or regional tumor involvement, thus influencing the surgical approach [24]. The laparoscopic approach was first proposed by Henry et al. for benign tumors under 4 cm and for non-invasive ones which do not exceed 12 cm [25]. This recommendation has greatly changed in recent decades, and, according to current ESES (European Society of Endocrine Surgeons) guidelines, the TP approach has been proven safe and oncologically sufficient for adrenocortical carcinomas not exceeding 10 cm [26]. Indeed, literature data reports successful removals of giant (> 10 cm), malignant lesions with the transperitoneal approach, which underlines the need for the expansion of indications [9]. In the surgical management of large, malignant adrenal tumors, oncological radicality, which maintains complete R0 resection, is of pivotal importance. Numerous studies support the choice of minimally invasive adrenalectomies in these cases, and, in terms of operative time and intraoperative blood loss, these studies report results that are at least comparable with the open method [9, 27]. The most time-consuming part in both methods is the dissection of tumor borders [28]. Analysis of the operative time-dependent efficacy showed that tumor size prolonged the operative time in TP cases, while this was true for tumor size and high BMI in RP cases [28]. In this respect, location and method of specimen removal may have affected the duration of surgery.

The dissected tumor may be retrieved through numerous locations, such as extended incisions of working ports (periumbilical, lateral), or through auxiliary incisions (pararectal, horizontal) and even a mini-Pfannenstiel incision. For TP, in cases involving large (6–10 cm) or extra-large (> 10 cm) adrenal lesions, we preferred the latter incision due to its excellent cosmesis and low rate of hernia development.

With regard to the presence of pheochromocytomas, minimally invasive removal of such lesions is often challenging. Numerous reports confirm that laparoscopic removal of pheochromocytomas can be carried out safely with a low morbidity rate [29–31]. However, in terms of the RP approach, the surgical indications for large pheochromocytomas should be further investigated [32, 33].

Among the patients analyzed, pheochromocytomas occurred in 9 TP cases (6.66%) and 5 RP cases (17.85%), possibly influencing the rate of conversion, resulting in 6 (4.44%) and 5 (18%) converted patients for TP and RP, respectively. Regarding the removal of pheochromocytomas and the frequency of conversions, no conversion to open surgery was necessary in the TP group, while 2 conversions (40%) occurred during RP resections. The mean size of pheochromocytomas was 63.85 mm and 39.8 mm for TP and RP, respectively. Increased conversion rates in RP may have occurred due to the surgically more challenging and thus prolonged LC, a high proportion of patients (*n* = 10; 35.71%) with a BMI ≥ 30 kg/m^2^, and a high number of lesions that turned out to be hormonally active pheochromocytomas (*n* = 5; 17.8%).

Although previous abdominal surgery was significantly higher for TP [*n* = 61 (45.18%); *p* = 0.038], interestingly, our study found a conversion rate of 4.44% and 18% for TP and RP, respectively, which indicates no disadvantage for TP in terms of conversions. A similar phenomenon has been confirmed by other work groups as well; i.e., there is no evident and direct connection between previous abdominal surgery/adhesion formation and increased number of conversions [34, 35].

Direct access to the adrenals without incursion into intraabdominal organs ranks as the most important advantage of RP adrenalectomies, although a strong possibility of injuries may arise during blind dissection of the lesion and vasculature, leading to potentially severe collateral damage. According to Munch et al., when the opening of the abdominal cavity is avoided, the rate of postoperative ileus, bacterial infection and intestine-related complications may be decreased in RP [36]. In our case, no postoperative ileus occurred after 135 TP adrenalectomies. In terms of perioperative complications, the two groups showed no significant difference. In terms of early complications (1–2 days after surgery), splenic injury, fever, severe hyperkalemia, intraoperative bleeding and death due to ventricular fibrillation occurred with TP, while severe wound infection and intraoperative bleeding occurred in 2-2 RP cases. As a late onset complication (12–21 months after surgery), postoperative abdominal hernia developed in one TP patient (Table 4).

Following the initial dominance of the RP approach, we have gradually switched to the TP technique in recent years, as indicated by the significantly higher number of TP cases. Our goal was to compare both methods, and TP proved to be superior in terms of previous abdominal surgery, mean tumor size and mean operative time.

The present analysis has certain limitations, which is mainly due to its retrospective nature and low number of patients; however, the relatively high patient accrual and the analysis of the learning curve may increase the scientific value of these results. In terms of the LC, we noted results that significantly favored the TP approach (previous abdominal surgery, conversion rate and operative time). Based on these, we conclude that the learning time for TP is much shorter, especially for surgeons familiar with laparoscopy.

## Conclusion

Both laparoscopic TP and RP adrenalectomies are safe and feasible, minimally invasive methods. According to our own results, RP adrenalectomy proved to be effective in the removal of smaller lesions, while TP adrenalectomy was shown to be more effective in the resection of large, extra-large and malignant lesions with a significantly shorter operative time. The analysis of the learning curve confirmed that TP can be carried out more rapidly, regardless of possible previous abdominal adhesions, thus resulting in lower conversion rates.

## References

[1] Gagner M, Pomp A, Heniford BT, Pharand D, Lacroix A (1997). Laparoscopic ad renalectomy lessons learned from 100 consecutive procedures. Ann Surg.

[2] Mercan S, Seven R, Ozarmagan S, Tezelman S (1995). Endoscopic retroperitoneal adrenalectomy. Surgery.

[3] Walz MK, Peitgen K, Krause U, Eigler FW (1995). Dorsal retroperitoneoscopic adrenalectomy—a new surgical technique. Zentralbl Chir.

[4] Walz MK, Alesina PF, Wenger FA, Koch JA, Neumann HP, Petersenn S, Schmid KW, Mann K (2006). Laparoscopic and retroperitoneoscopic treatment of pheochromocytomas and retroperitoneal paragangliomas: results of 161 tumors in 126 patients. World J Surg.

[5] Lee CR, Walz MK, Park S, Park JH, Jeong JS, Lee SH, Kang SW, Jeong JJ, Nam KH, Chung WY, Park CS (2012). A comparative study of the transperitoneal and posterior retroperitoneal approaches for laparoscopic adrenalectomy for adrenal tumors. Ann Surg Oncol.

[6] Mohammadi-Fallah MR, Mehdizadeh A, Badalzadeh A, Izadseresht B, Dadkhah N, Barbod A, Babaie M, Hamedanchi S (2013). Comparsion of transperitoneal versus retroperitoneal laparoscopic adrenalectomy in a prospective randomized study. J Laparoendosc Adv Surg Tech A.

[7] Tiberio GA, Solaini L, Arru L, Merigo G, Baiocchi GL, Giulini SM (2013). Factors influencing outcomes in laparoscopic adrenal surgery. Langenbecks Arch Surg.

[8] Mege D, Taieb D, Lowery A, Loundou A, De Micco C, Castinetti F, Morange I, Henry JF, Sebag F (2014). Contemporary review of large adrenal tumors in a tertiary referral center. Anticancer Res.

[9] Ottlakán A, Paszt A, Borda B, Simonka Z, Ábrahám S, Lázár G (2017). Removal of giant adrenal tumors using the laparoscopic transperitoneal technique. A report of three successful cases. Orv Hetil.

[10] Shonkwiler RJ, Lee JA (2011). Laparoscopic retroperitoneal adrenalectomy. Surg Laparosc Endosc Percutan Tech.

[11] Nigri G, Rosman AS, Petrucciani N, Fancellu A, Pisano M, Zorcolo L, Ramacciato G, Melis M (2013). Meta-analysis of trials comparing laparoscopic transperitoneal and retroperitoneal adrenalectomy. Surgery.

[12] Bonjer HJ, van der Harst E, Steyerberg EW, de Herder WW, Kazemier G, Mohammedamin RS, Bruining HA (1998). Retroperitoneal adrenalectomy: open or endoscopic?. World J Surg.

[13] Berber E, Tellioglu G, Harvey A, Mitchell J, Milas M, Siperstein A (2009). Comparison of laparoscopic transabdominal lateral versus posterior retroperitoneal adrenalectomy. Surgery.

[14] Chen Y, Scholten A, Chomsky-Higgins K, Nwaogu I, Gosnell JE, Seib C, Shen WT, Suh I, Duh QY (2018). Risk factors associated with perioperative complications and prolonged length of stay after laparoscopic adrenalectomy. JAMA Surg.

[15] Balogh Á, Varga L, Julesz J, Lázár G, Martin K (2000). Minimally invasive adrenalectomy with posterior retroperitoneoscopy. Orv Hetil.

[16] Lee J, El-Tamer M, Schifftner T, Turrentine FE, Henderson WG, Khuri S, Hanks JB (2008). Open and laparoscopic adrenalectomy: analysis of the National Surgical Quality Improvement Program. J Am Coll Surg.

[17] Hasegawa M, Miyajima A, Jinzaki M, Maeda T, Takeda T, Kikuchi E, Shibata H, Oya M (2013). Visceral fat is correlated with prolonged operative time in laparoendoscopic single-site adrenalectomy and laparoscopic adrenalectomy. Urology.

[18] Constantinides VA, Christakis I, Touska P, Meeran K, Palazzo F (2013). Retroperitoneoscopic or laparoscopic adrenalectomy? A single-centre UK experience. Surg Endosc.

[19] Elfenbein DM, Scarborough JE, Speicher PJ, Scheri RP (2013). Comparison of laparoscopic versus open adrenalectomy: results from American College of Surgeons-National Surgery Quality Improvement Project. J Surg Res.

[20] Conzo G, Musella M, Corcione F, De Palma M, Ferraro F, Palazzo A, Napolitano S, Milone M, Pasquali D, Sinisi AA, Colantuoni V, Santini L (2013). Laparoscopic adrenalectomy, a safe procedure for pheochromocytoma. A retrospective review of clinical series. Int J Surg.

[21] Shen ZJ, Chen SW, Wang S, Jin XD, Chen J, Zhu Y, Zhang RM (2007). Predictive factors for open conversion of laparoscopic adrenalectomy: a 13-year review of 456 cases. J Endourol.

[22] Zonča P, Bužga M, Ihnát P, Martínek L (2015). Retroperitoneoscopic adrenalectomy in obese patients: is it suitable?. Obes Surg.

[23] Shiraishi K, Kitahara S, Ito H, Oba K, Ohmi C, Matsuyama H (2019). Transperitoneal versus retroperitoneal laparoscopic adrenalectomy for large pheochromocytoma: comparative outcomes. Int J Urol.

[24] Gumbs AA, Gagner M (2006). Laparoscopic adrenalectomy. Best Practice & Research. Clin Endocrinol Metabol.

[25] Henry JF, Defechereux T, Gramatica L, Raffaelli M (1999). Should laparoscopic approach be proposed for large and/or potentially malignant adrenal tumors?. Langenbecks Arch Surg.

[26] Henry JF, Peix JL, Kraimps JL (2012). Positional statement of the European Society of Endocrine Surgeons (ESES) on malignant adrenal tumors. Langenbecks Arch Surg.

[27] Dalvi AN, Thapar PM, Thapar VB, Rege SA, Deshpande AA (2012). Laparoscopic adrenalectomy for large tumours: single team experience. J Minim Access Surg.

[28] Berber E, Duh QY, Clark OH, Siperstein AE (2002). A critical analysis of intraoperative time utilization in laparoscopic adrenalectomy. Surg Endosc.

[29] Kercher KW, Novitsky YW, Park A, Matthews BD, Litwin DE, Heniford BT (2005). Laparoscopic curative resection of pheochromocytomas. Ann Surg.

[30] Perry KA, El Youssef R, Pham TH, Sheppard BC (2010). Laparoscopic adrenalec-tomy for large unilateral pheochromocytoma: experience in a large academicmedical center. Surg Endosc.

[31] Boylu U, Oommen M, Lee BR, Thomas R (2009). Laparoscopic adrenalectomy forlarge adrenal masses: pushing the envelope. J Endourol.

[32] Wang B, Ma X, Li H, Shi T, Hu D, Fu B, Lang B, Chen G, Zhang X (2011). Anatomic retroperitoneoscopic adrenalectomy forseleted adrenal tumors >5 cm: our technique and experience. Urology.

[33] de Fourmestraux A, Salomon L, Abbou CC, Grise P (2015). Ten year experience of retroperitoneal laparoscopic resection for pheochromocytomas: a dual-centrestudy of 72 cases. World J Urol.

[34] Mazeh H, Froyshteter AB, Wang TS, Amin AL, Evans DB, Sippel RS, Chen H, Yen TW (2012). Is previous same quadrant surgery a contraindication to laparoscopic adrenalectomy?. Surgery.

[35] Economopoulos KP, Phitayakorn R, Lubitz CC, Sadow PM, Parangi S, Stephen AE, Hodin RA (2016). Should specific patient clinical characteristics discourage adrenal surgeons from performing laparoscopictransperitoneal adrenalectomy?. Surgery.

[36] Munch LC, Gill IS, McRoberts JW (1994). Laparoscopic retroperitoneal renal cystectomy. J Urol.

